# Myo-Inositol for Cutaneous Manifestations of Polycystic Ovary Syndrome: An International Delphi Consensus Recommendation for Dermatology Practice

**DOI:** 10.7759/cureus.111393

**Published:** 2026-06-23

**Authors:** Anil Ganjoo, Sanjay Kalra, Malavika Kohli, Rajetha Damisetty, Sachin Verma, Rajat Kandhari, Balram Sharma, Nilakshi Deka, Anne Beatrice, Ambika R Pradhan, Sudip Parajuli, Haikal A Rahman, Sarker Mahbub A Shamim, Rathish Nair, Pratik Patel, Girish R Kulkarni

**Affiliations:** 1 Dermatology, Skinnovation Clinics, New Delhi, IND; 2 Endocrinology, Bharti Hospital, Karnal, IND; 3 Dermatology, Breach Candy Hospital, Jaslok Hospital and Skin Secrets, Mumbai, IND; 4 Dermatology, Dr. Rajetha's Mohana Skin AND Hair Clinic, Hyderabad, IND; 5 Dermatology, Skinvita Clinic, Kolkata, IND; 6 Dermatology, Dr. Kandhari’s Skin and Dental Clinic, New Delhi, IND; 7 Endocrinology, SMS Medical College, Jaipur, IND; 8 Endocrinology, Diabetes and Metabolism, Apollo Hospitals, Guwahati, IND; 9 Endocrinology, Nizams Institute of Medical Sciences, Hyderabad, IND; 10 Dermatology, Jigme Dorji Wangchuck National Referral Hospital, Thimphu, BTN; 11 Dermatology, Institute of Medicine, Kathmandu, NPL; 12 Dermatology, Indira Gandhi Memorial Hospital, Male, MDV; 13 Dermatology and Venereology, MH Samorita Hospital and Medical College, Dhaka, BGD; 14 Medical Affairs, Torrent Pharmaceuticals Ltd., Ahmedabad, IND

**Keywords:** dermatological manifestation, folic acid, hormonal imbalance, myo-inositol, polycystic ovarian syndrome, vitamin d3

## Abstract

Purpose: Acne and hirsutism are among the most distressing features of polycystic ovary syndrome (PCOS), yet current guidelines largely emphasize metabolic and reproductive aspects, offering limited guidance for dermatologic management. Myo-inositol (MI), an insulin-sensitizing agent with endocrine benefits, has emerged as a promising nonhormonal option; however, its role in managing cutaneous symptoms remains unclear. The purpose of this study was to develop expert consensus on the use of MI alone and in combination with folic acid and vitamin D₃ for PCOS-related dermatologic manifestations.

Methods: Eleven statements were generated through a rigorous literature review and subsequently evaluated by 133 experts through a two-round online Delphi process. Consensus was defined as ≥70% agreement; statements with 50% to <70% agreement were revised and reassessed.

Results: Consensus endorsed MI as an effective and well-tolerated intervention for acne and hirsutism. Continuous MI supplementation at 4 g/day for ≥6 months was recommended. MI + folic acid + vitamin D₃ and MI + D-chiro-inositol were favored for metabolic dysfunction and hyperandrogenism.

Conclusion: MI-based therapy, particularly early initiation and strategic combinations, should be integrated into multimodal management of PCOS-related cutaneous manifestations.

## Introduction

Polycystic ovary syndrome (PCOS) is a common endocrine disorder affecting women of reproductive age, with symptoms often beginning in adolescence and fluctuating throughout reproductive life [1]. According to the World Health Organization, PCOS is characterized by hormonal imbalance, irregular menstrual cycles, biochemical or clinical hyperandrogenism, and polycystic ovarian morphology [2]. Global prevalence ranges from 4% to 21% depending on diagnostic criteria and, in India, has been reported between 7.2% and 19.6% using NIH (National Institutes of Health) 1990 and Rotterdam 2003 criteria [3]. Beyond reproductive dysfunction, PCOS is strongly associated with insulin resistance, obesity, type 2 diabetes mellitus, and cardiometabolic disease [2].

Dermatologic manifestations are highly prevalent and often serve as early clinical indicators, including acne, hirsutism, androgenetic alopecia, acanthosis nigricans, and striae [4]. Approximately 20%-40% of women with persistent or treatment-resistant acne may have underlying PCOS, while up to 43% of women diagnosed with PCOS experience acne [5], underscoring the close relationship between cutaneous symptoms and underlying endocrine dysfunction.

Hyperandrogenism, primarily driven by dysregulated steroidogenesis and exacerbated by elevated luteinizing hormone and hyperinsulinemia, results in excessive testosterone and dihydrotestosterone (DHT) production in ovarian theca cells [6-8]. These androgens stimulate sebum production, alter keratinocyte differentiation, and enhance 5α-reductase activity, leading to inflammatory acne [9], while promoting transformation of vellus hairs into coarse terminal hairs in androgen-sensitive body areas, resulting in hirsutism [10]. Despite the major psychosocial impact of such manifestations, the absence of standardized dermatologic management approaches has resulted in inconsistent treatment outcomes [11].

Myo-inositol (MI), a naturally occurring insulin-sensitizing molecule, has gained significant attention for its role in PCOS management. MI participates in insulin signaling and ovarian follicular physiology [12] and has been shown to improve metabolic and hormonal abnormalities, reduce circulating testosterone, and enhance ovulatory function [13]. Recent clinical evidence also suggests that MI may improve cutaneous symptoms of PCOS, such as acne and hirsutism, potentially broadening its therapeutic value in aesthetic and endocrine dermatology [14-18]. However, lack of clarity regarding optimal dosing, duration, therapeutic combinations, and clinical positioning of MI continues to limit uniform adoption in gynecology and dermatology practice [19].

To address this gap, the Delphi consensus aimed to define evidence-based recommendations on the role of MI alone or in combination with folic acid (FA) and vitamin D3 in the management of PCOS-associated cutaneous manifestations. The process established guidance on indications, dosing, duration, efficacy, and safety to support consistent clinical decision-making. The Hirsutism and Acne Lowering Treatment-South Asian Association for Regional Cooperation-Association of Aesthetic Dermatology (HALT SAARC-AAD) Study Group, comprising dermatology experts from India and neighboring SAARC countries, ensured that the recommendations reflected both scientific evidence and real-world practice patterns across South Asia.

## Materials and methods

Literature review and search strategy

A comprehensive literature review was conducted (January-March 2024) to evaluate current evidence on the role of MI in managing cutaneous manifestations of PCOS. Searches were performed in Web of Science, PubMed, Embase, Cochrane Library, and Google Scholar using MeSH and free-text terms related to PCOS, MI, insulin resistance, hyperandrogenism, and dermatologic outcomes. Randomized controlled trials, meta-analyses, and observational studies published in English from 2015 to 2024 were included. Evidence synthesis focused on MI mechanisms, clinical efficacy for acne, hirsutism, and androgenetic alopecia, optimal dose and duration, and therapeutic roles of combination regimens.

Expert scientific committee and statement development

Following the literature review, a structured knowledge, attitude, and practices (KAP) questionnaire was developed in April 2024 to evaluate clinical practices related to MI for PCOS-associated cutaneous manifestations. A scientific committee of 13 experts (9 dermatologists and 4 endocrinologists) from SAARC nations and India, under HALT SAARC-AAD, reviewed the questionnaire and guided consensus development. These experts were selected based on 15-20 years of experience in PCOS management, at least three indexed publications, and prior scientific speaking experience. The panel developed 11 evidence-based statements outlining MI's role, suitable combinations, and recommended dosing for dermatologic manifestations in PCOS. HALT SAARC-AAD was the coordinating body for the panel process and not an entity influencing the independent clinical judgments of the experts.

Delphi study design and panel recruitment

An online Delphi process was conducted to achieve consensus on the proposed statements. Two authors (RN, AG) acted as Delphi coordinators and were responsible for survey development, distribution of invitations, monitoring responses, reminders, and data management across rounds. An international panel of experts was recruited based on clinical experience in PCOS and familiarity with MI use. A total of 150 healthcare professionals were approached via email and virtual meetings, supported by four zonal and international meetings held between June and August 2024. Experts were given four weeks to confirm participation, with two reminders issued during each round.

Rating process and consensus criteria

The Delphi survey was administered electronically, and each healthcare professional independently rated agreement with the proposed clinical statements on a five-point Likert scale (1 = completely disagree; 5 = completely agree). Responses were anonymized to minimize bias. An open-ended comment box accompanied each item to obtain qualitative feedback and wording recommendations. A predefined agreement level of ≥70% was set for consensus and statement acceptance, whereas ≤50% indicated rejection. Statements scoring between 50% and <70% underwent refinement or were rejected by the expert scientific committee, and refined statements were re-evaluated by the same panel in subsequent rounds to achieve consensus.

Delphi rounds

**Figure 1:**
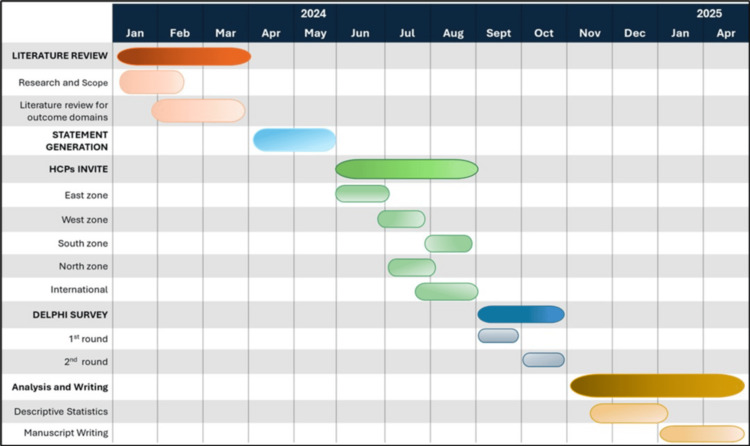
Delphi process timeline flow chart Project timeline for the Delphi consensus process on PCOS-related cutaneous manifestations, illustrating the sequential phases of literature review, statement generation, healthcare professional invitations across geographic zones, Delphi survey rounds, and analysis and manuscript preparation. The figure was created using Microsoft PowerPoint (Microsoft Corporation, Redmond, Washington).

The first Delphi round was conducted in September 2024, during which panelists rated 11 statements using a five-point Likert scale. Statements with <50% agreement were excluded, while those with 50% to <70% agreement were reconsidered based on qualitative feedback. An advisory board meeting of the steering committee was held between rounds to review responses and refine statements, resulting in four updated items for the second round. The second online Delphi round was conducted in October 2024, during which four statements requiring reassessment were rated using the same Likert scale. Following completion of round two, the steering committee reviewed all responses and finalized the consensus recommendations (Figure 1). All invited panelists participated throughout the consensus process, and no attrition was observed.

Descriptive statistics

Descriptive statistics were applied to summarize expert responses across all rounds. In each round, the frequency, percentage, median, and interquartile range (IQR) were calculated using R software (Version 4.5.2, R Foundation for Statistical Computing, Vienna, Austria). The median values were 3 or above, and the IQRs were 1 or less, reflecting strong consensus among the expert panel [20].

Ethics approval

As this was a Delphi consensus study, formal review or approval by an ethics committee was not required.

## Results

**Figure 2:**
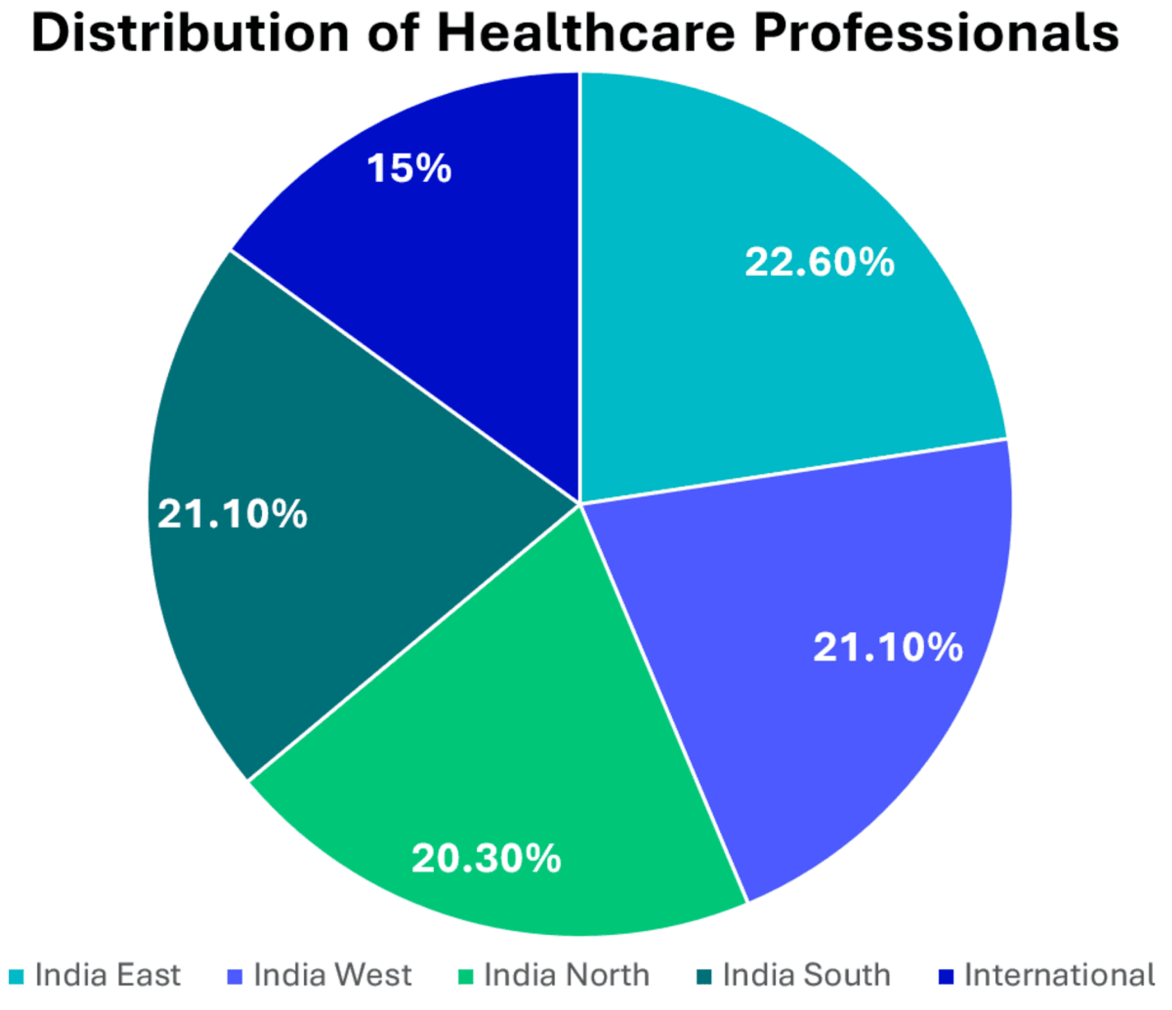
Distribution of panelists by zone Distribution of participating healthcare professionals, showing that 85% were practicing in India and 15% were from international centres. The figure was created using Microsoft Excel (Microsoft Corporation, Redmond, Washington).

**Figure 3:**
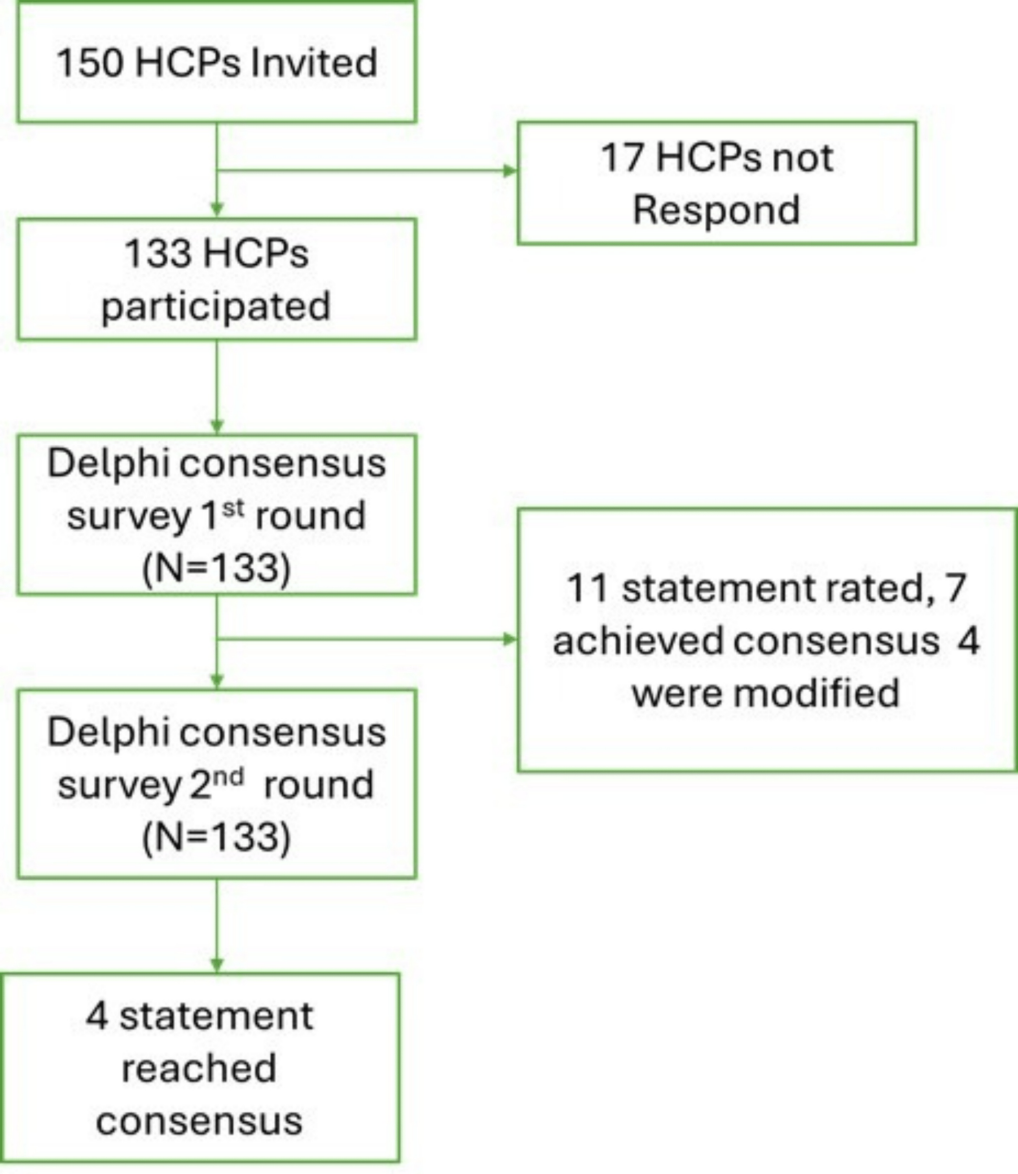
Flow of panelists participation leading to consensus Flow of healthcare professionals through the Delphi process: 150 HCPs were invited, 133 participated in round 1 and 2, and a total of 11 statements reached consensus. The figure was created using Microsoft PowerPoint (Microsoft Corporation, Redmond, Washington).

Of the 150 invited panelists, 133 participated in the Delphi process, including 113 experts from India (representing the east, west, south, and north zones) and 20 from neighboring SAARC countries. All participants were experienced dermatologists (Figures 2, 3). Of the 11 initial statements, seven achieved consensus, and four were modified during a steering committee meeting (Tables 1, 2). 

**Table 1:** Primary eleven "consensus statements" with agreement numbers in the Delphi round 1 OCPs: oral contraceptive pills, PCOS: polycystic ovary syndrome, IQR: interquartile range.

Item	Statements	Agree (N)	Median	IQR	Percent Agreement	Consensus
1	Although OCPs are the first-line treatment with PCOS, they only treat the hyperandrogenism symptoms of the syndrome, without addressing ovarian issues or metabolic alterations	80	3	1	60.6	Statement Modified
2	Myo-inositol administration is an effective therapeutic option in women with PCOS, as it improves insulin resistance and reduces hyperandrogenism	122	3	0	92.4	Consensus Achieved
3	The therapeutic response to myo-inositol is more pronounced in hyperandrogenic PCOS phenotypes than in non-hyperandrogenic phenotypes	91	3	1	70	Consensus Achieved
5	Myo-inositol supplementation is associated with a reduction in the severity of hirsutism and acne in women with PCOS	113	3	0	86.3	Consensus Achieved
6	Cutaneous manifestations of PCOS, including acne and hirsutism, should be managed using a multimodal approach integrating myo-inositol, hormonal regulation, and dermatological therapies	99	3	0	76.2	Consensus Achieved
7	Initiating myo-inositol therapy early in PCOS, particularly at the onset of acne or hirsutism, is beneficial for optimizing cutaneous outcomes	100	3	0	76.9	Consensus Achieved
8	A dosage of 4 g of myo-inositol per day is effective in improving cutaneous symptoms such as acne and hirsutism in PCOS	83	3	2	62.4	Statement Modified
9	A minimum treatment duration of 6 months with myo-inositol is necessary to obtain meaningful clinical benefits in PCOS-related acne and hirsutism	76	3	2	59.8	Statement Modified
10	A daily combination of myo-inositol and D-chiro-inositol is the preferred supplementation for effective management of PCOS metabolic and dermatological symptoms	85	3	2	65.4	Statement Modified
11	The combination of myo-inositol, folic acid, and vitamin D3 is a safe and effective primary treatment for reducing PCOS-related symptoms and dermatological manifestations	126	3	0	94.7	Consensus Achieved

**Table 2:** Four modified "consensus statements" with agreement numbers in the Delphi round 2 IQR: interquartile range, PCOS: polycystic ovary syndrome.

Item	Statements	Agree (N)	Median	IQR	Percent Agreement	Consensus
1	The recommended dosage of myo-inositol for the treatment of cutaneous manifestations, including acne and hirsutism, in PCOS is 4 g per day	101	3	0	75.9	Consensus Achieved
2	Six months of continuous myo-inositol supplementation significantly improves cutaneous manifestations, including acne and hirsutism, in patients with PCOS	103	3	0	77.4	Consensus Achieved
3	The combination of myo-inositol and D-chiro-inositol is more effective in controlling hyperandrogenism and other symptoms of PCOS than myo-inositol alone	113	3	0	85	Consensus Achieved
4	Myo-inositol is considered a first-line treatment for managing cutaneous manifestations, such as hirsutism and acne, in patients with PCOS	103	3	0	77.4	Consensus Achieved

## Discussion

This Delphi consensus provides expert-driven recommendations on the role of MI in the management of cutaneous manifestations associated with PCOS. While international PCOS guidelines primarily focus on metabolic, endocrine, and reproductive aspects, guidance for dermatologic concerns such as acne, hirsutism, and androgenetic alopecia remains limited in scope and lacks clear therapeutic direction despite their significant impact on patients' quality of life. By synthesizing evidence from randomized controlled trials, meta-analyses, and observational studies and integrating multidisciplinary expert opinion through a structured two-round Delphi process, this consensus addresses an important gap in clinical practice. The iterative refinement of statements and broad representation of dermatologists and endocrinologists from SAARC nations enhance the robustness and real-world applicability of these recommendations across diverse clinical settings.

Based on the steering committee expert panel's evaluation of clinical and scientific data, key recommendations are discussed below.

Statement 1

The combination of MI, FA, and vitamin D3 is a safe and effective primary treatment for reducing PCOS-related symptoms and dermatologic manifestations.

The consensus supporting the combination of MI, FA, and vitamin D₃ as a safe and effective primary treatment for reducing PCOS-related symptoms and dermatologic manifestations aligns well with published evidence. A prospective study using a proprietary MI + FA + vitamin D₃ formulation reported substantial reductions in acne lesions, improvement in hirsutism scores, and menstrual regularization with negligible adverse effects [14]. Vitamin D deficiency is prevalent in PCOS and contributes to insulin resistance, inflammation, and androgen excess; co-supplementation with vitamin D may therefore potentiate MI-mediated metabolic and cutaneous benefits [21]. A systematic review indicated that combining inositols with micronutrients such as FA and vitamin D may offer broader endocrine and dermatologic improvements than inositols alone [22]. A recent review by Quach et al. (2024) further supported the therapeutic value of inositol for both PCOS-related and non-PCOS-related acne and hirsutism [23]. The high level of agreement among the SAARC-AAD expert panel underscores its relevance in South Asian practice, where nonhormonal and well-tolerated therapies are commonly preferred.

Statement 2

The combination of MI and D-chiro-inositol is more effective in controlling hyperandrogenism and other symptoms of PCOS than MI alone.

MI and D-chiro-inositol (DCI) are stereoisomers that regulate insulin signaling, ovarian steroidogenesis, and glucose metabolism. While MI alone improves insulin sensitivity and ovulatory function, combining MI and DCI in the physiological 40:1 ratio has demonstrated superior outcomes in women with PCOS, especially those with metabolic dysfunction or marked hyperandrogenism. Randomized trials show greater reductions in androgen levels and enhanced metabolic and ovulatory profiles with MI + DCI compared with MI alone [24, 25], and meta-analyses confirm higher ovulation rates and improved insulin resistance with low-dose DCI alongside MI [17, 26]. The 40:1 ratio reflects healthy follicular fluid composition and prevents the androgen-stimulating effects seen with high-dose DCI [25]. However, the clinical benefits appear to be strongly dependent on the MYO:DCI ratio, and the optimal proportion remains uncertain. Inaki et al. reported that the commonly used 40:1 ratio is derived primarily from plasma physiology rather than follicular physiology and emphasized that higher MYO-dominant ratios (e.g., >40:1 up to 100:1) warrant further investigation. Additionally, DCI administered alone, particularly at higher doses, has not demonstrated consistent clinical benefit, and increasing the proportion of DCI may adversely affect ovulation and reproductive outcomes [26].

Statement 3

The recommended dosage of MI for the treatment of cutaneous manifestations, including acne and hirsutism, in PCOS, is 4 g/day.

A daily dosage of 4 g MI, typically administered as 2 g twice daily, is widely recognized as the optimal regimen for improving symptoms of PCOS. This dose provides sufficient circulating levels to achieve insulin-sensitizing activity and support ovarian function. Recent guidance from the Society of Obstetricians and Gynaecologists of Canada identifies MI as a safe insulin-sensitizing option, noting that most clinical studies have used 4 g/day and that this dose is consistently associated with superior reproductive and metabolic outcomes compared with lower doses [27].

Observational data further show improvements in menstrual regularity, insulin resistance, ovulation, and hyperandrogenic manifestations such as hirsutism and acne [28]. A systematic review by Genazzani et al. (2016) also demonstrated improved insulin resistance and normalization of the LH/FSH ratio after MI therapy [13].

Statement 4

Six months of continuous MI supplementation significantly improves cutaneous manifestations, including acne and hirsutism, in patients with PCOS.

MI, a key mediator in insulin signaling, has shown clinically meaningful benefits in improving dermatologic manifestations of PCOS, particularly acne and hirsutism. Sustained therapy appears essential for optimal results. Qamar et al. (2020) demonstrated that six-month MI therapy significantly improved acne and hirsutism in young women with PCOS [29]. Similarly, Malik et al. (2021) reported that three to six months of MI supplementation enhanced menstrual regularity, insulin resistance, ovulation, and hyperandrogenic features, including acne and hirsutism [28]. Genazzani et al. (2008) and another study observed marked reductions in acne severity and circulating androgen levels after approximately 24 weeks of treatment, indicating a time-dependent therapeutic effect [13, 15]. Collectively, these findings support continuous MI use for at least six months to achieve meaningful dermatologic benefit.

Comparative clinical trials further indicate that MI offers efficacy similar to metformin in improving insulin resistance and hormonal parameters, but with superior tolerability. In a six-month randomized trial, MI produced significantly fewer adverse effects than metformin despite comparable metabolic outcomes [30]. Likewise, Bodepudi et al. (2023) reported no significant difference in metabolic or androgenic outcomes over 24 weeks, reinforcing MI as a well-tolerated first-line alternative for patients who experience gastrointestinal intolerance to metformin [31].

Statement 5

MI is considered a first-line treatment for managing cutaneous manifestations, such as hirsutism and acne, in patients with PCOS.

Given its favorable safety profile and metabolic benefits, MI is increasingly viewed as a first-line therapy for dermatologic manifestations of PCOS. Unlike oral contraceptives or anti-androgens, MI targets underlying insulin resistance and hyperandrogenism, offering a disease-modifying approach. Zacchè et al. demonstrated that six months of MI supplementation significantly reduced acne severity and hirsutism in young women with PCOS, along with improvements in hormonal balance [15]. Qamar and Mustafa similarly reported reductions in cutaneous symptoms and metabolic parameters following MI therapy [29]. A meta-analysis by Unfer et al. confirmed that MI lowers circulating androgen levels and improves insulin sensitivity pathways that drive androgen-mediated dermatologic symptoms [16]. Studies by Lagana et al. (2015) and Garg et al. (2016) also emphasize that early MI initiation improves insulin sensitivity and reduces hyperandrogenism, potentially preventing or attenuating acne and hirsutism [32, 33].

In summary, the current Delphi consensus, supported by clinical evidence (Appendices), identifies MI as a safe and effective treatment for PCOS-related cutaneous manifestations. A regimen of 4 g/day for at least six months, preferably combined with folic acid and vitamin D₃, is strongly recommended to achieve optimal dermatologic outcomes.

However, this Delphi consensus is limited by its reliance on expert opinion, which is subject to inherent methodological constraints, including respondent subjectivity and the potential influence of prevailing clinical perspectives. The conclusions are derived from the interpretation of existing literature and clinical experience, in the absence of dedicated prospective randomized outcome trials.

## Conclusions

This Delphi consensus by the HALT SAARC-AAD Study Group reflects expert agreement supporting MI as an effective and well-tolerated first-line therapeutic option for the management of PCOS-related cutaneous manifestations. Based on collective panel opinion and available evidence, a daily dose of 4 g administered for 6 months was considered an appropriate regimen to address underlying metabolic and hormonal abnormalities associated with PCOS, including insulin resistance and hyperandrogenism. These consensus statements provide guidance for clinical practice; however, future well-designed prospective and long-term studies are required to validate these recommendations, assess sustained outcomes, and explore combination approaches to further optimize PCOS management.

## Appendices

**Table 3:** Evidence review OCP: oral contraceptive pills, MI: myo-inositol, DCI: D-chiro inositol, PCOS: polycystic ovarian syndrome, LH: luteinizing hormone, FSH: follicle-stimulating hormone.

Citation	Study Design	Study Population	Study Drug	Duration of Therapy	Dose of MI	Results and Conclusion
Myo-inositol for cutaneous manifestation						
*Eur Rev Med Pharmacol Sci*. 2021;25(23):7476-7485. doi:10.26355/eurrev_202112_27447	Randomized controlled trial	13-16 and 17-19 years old	OCP vs MI vs (OCP+MI)	3 months	4 g/d	Therapy based on this natural compound, alone or in combination with OCP, seems effective in improving both metabolic and hormonal parameters in PCOS women and thereby reducing acne.
*Eur Rev Med Pharmacol Sci*. 2019;23(12):5512-5521. doi:10.26355/eurrev_201906_18223	Randomized controlled trial	56	MI/DCI	3 months	4 g/d	The 40:1 MI/DCI ratio is the best for PCOS therapy aimed at restoring ovulation and normalizing skin manifestations in these patients.
*Pak J Med Health Sci*. 2022;16(1):21-24. doi:10.53350/pjmhs2216121	Prospective cohort study	106	(MI+DCI) vs Placebo	6 months	1.1 g/d (40:1 ratio)	A statistically significant improvement was observed in clinical & Laboratory parameters in the treatment group, showing reduced LH:FSH ratio, fasting glucose, and increased Estradiol levels, along with decreased BMI, Acne, and hair growth, and improved ultrasound parameters in the same group as compared to the placebo group.
*Clin Endocrinol (Oxf)*. 2023;99(2):198-205. doi:10.1111/cen.14931	Randomized controlled trial	53	(MI + Metformin) vs Metformin alone	6 months	Not available	The addition of myoinositol to metformin exerts additional benefits in improving menstrual cycle regularity and improving skin manifestation and quality of life in women with PCOS.
*Gynecol Endocrinol*. 2019;35(6):511-514. doi:10.1080/09513590.2018.1549656	Randomized controlled trial	120	(MI + Metformin) vs Metformin alone	3 months	1.8 g/d	The biochemical and hormonal parameters were improved in Metformin + myo-inositol as compared to Metformin alone. Cutaneous manifestations were reduced for patients on the combination therapy.
*Minerva Ginecol*. 2015;67(4):321-325	Randomized controlled trial	137	MI vs DCI vs Placebo	6 months	Not available	Both myo-inositol (MI-PG) and D-chiro inositol (DCI-PG) treatments were able to significantly improve the regularity of the menstrual cycle, the Acne Score, the endocrine and metabolic parameters and the insulin-resistance in young, overweight, PCOS patients.
*J Endocrinol Invest*. 2022;45(3):583-595. doi:10.1007/s40618-021-01691-5	Randomized controlled trial	66	MI vs Metformin	6 months	4 g/d	Study showed similar effects of MET and MI on BMI, hormonal profile, acne control, metabolism of glucose and insulin, and adiponectin level.
Myo-inositol for hyperandrogenism and insulin resistance						
*Iraqi J Pharm Sci*. 2023;32(Suppl):61-73. doi:10.31351/vol32issSuppl.pp61-73	Randomized open-label, prospective study	54	MI vs Metformin vs (MI+Metformin)	3 months	4 g/d	Combining Myoinositol plus metformin, along with lifestyle adherence, improves clinical, endocrine, and metabolic parameters, besides oxidative markers in PCOS women. Compared to metformin, Myoinositol has greater patient compliance, is more well tolerated, and is thought to be the best medication to complement existing standard therapies with a significant effect on insulin resistance and androgen levels.
*Al-Azhar Int Med J*. 2024;5(7):7. doi:10.58675/2682-339X.2526	Prospective observational cohort study	200	Myo-inositol	6 months	4 g/d	For PCOS women, Myo-inositol treatment is a safe, efficient method of ovulation induction and conception. It resulted in considerable restoration of spontaneous ovarian activity and improvement in pregnancy rate.
*Int J Gynaecol Obstet*. 2022;158(2):278-284. doi:10.1002/ijgo.13971	Randomized open-label study	70	(MI + DCI) vs CHC	6 months	4 g/d	Myo-inositol and d-chiro-inositol in combination (3.6:1 ratio) are effective in regularizing menstrual cycles and improving insulin resistance.

## References

[1] Ding DC, Chen W, Wang JH, Lin SZ (2018). Association between polycystic ovarian syndrome and endometrial, ovarian, and breast cancer: a population-based cohort study in Taiwan. Medicine (Baltimore).

[2] (2025). Polycystic ovary syndrome. https://www.who.int/news-room/fact-sheets/detail/polycystic-ovary-syndrome.

[3] Ganie MA, Chowdhury S, Malhotra N (2024). Prevalence, phenotypes, and comorbidities of polycystic ovary syndrome among Indian women. JAMA Netw Open.

[4] Keen MA, Shah IH, Sheikh G (2017). Cutaneous manifestations of polycystic ovary syndrome: a cross-sectional clinical study. Indian Dermatol Online J.

[5] Ramezani Tehrani F, Behboudi-Gandevani S, Bidhendi Yarandi R, Saei Ghare Naz M, Carmina E (2021). Prevalence of acne vulgaris among women with polycystic ovary syndrome: a systemic review and meta-analysis. Gynecol Endocrinol.

[6] Archer JS, Chang RJ (2004). Hirsutism and acne in polycystic ovary syndrome. Best Pract Res Clin Obstet Gynaecol.

[7] Dong J, Rees DA (2023). Polycystic ovary syndrome: pathophysiology and therapeutic opportunities. BMJ Med.

[8] Housman E, Reynolds RV (2014). Polycystic ovary syndrome: a review for dermatologists: Part I. Diagnosis and manifestations. J Am Acad Dermatol.

[9] Thiboutot D (2004). Acne: hormonal concepts and therapy. Clin Dermatol.

[10] Rosenfield RL (2005). Clinical practice. Hirsutism. N Engl J Med.

[11] Teede HJ, Tay CT, Laven JJ (2023). Recommendations from the 2023 international evidence-based guideline for the assessment and management of polycystic ovary syndrome. J Clin Endocrinol Metab.

[12] Croze ML, Soulage CO (2013). Potential role and therapeutic interests of myo-inositol in metabolic diseases. Biochimie.

[13] Genazzani AD, Lanzoni C, Ricchieri F, Jasonni VM (2008). Myo-inositol administration positively affects hyperinsulinemia and hormonal parameters in overweight patients with polycystic ovary syndrome. Gynecol Endocrinol.

[14] Ramanan EA, Ravi S, Anbu KR, Michael M (2020). Efficacy and safety of Tracnil™ administration in patients with dermatological manifestations of PCOS: an open-label single-arm study. Dermatol Res Pract.

[15] Zacchè MM, Caputo L, Filippis S, Zacchè G, Dindelli M, Ferrari A (2009). Efficacy of myo-inositol in the treatment of cutaneous disorders in young women with polycystic ovary syndrome. Gynecol Endocrinol.

[16] Unfer V, Nestler JE, Kamenov ZA, Prapas N, Facchinetti F (2016). Effects of Inositol(s) in women with PCOS: a systematic review of randomized controlled trials. Int J Endocrinol.

[17] Unfer V, Facchinetti F, Orrù B, Giordani B, Nestler J (2017). Myo-inositol effects in women with PCOS: a meta-analysis of randomized controlled trials. Endocr Connect.

[18] Pundir J, Psaroudakis D, Savnur P (2018). Inositol treatment of anovulation in women with polycystic ovary syndrome: a meta-analysis of randomised trials. BJOG.

[19] Moretti C, Bonomi M, Dionese P (2024). Inositols and female reproduction disorders: a consensus statement from the working group of the Club of the Italian Society of Endocrinology (SIE)-Women's Endocrinology. J Endocrinol Invest.

[20] Chang YC, Chuang RS, Hsiao CT, Khwepeya M, Nkambule NS (2022). Bridging the gap: using consensus to explore entrustment decisions and feedback receptivity in competency-based emergency medicine residency programs through the construction of a Q-sample incorporating a Delphi technique. Front Med (Lausanne).

[21] Katyal G, Kaur G, Ashraf H, Bodapati A, Hanif A, Okafor DK, Khan S (2024). Systematic review of the roles of inositol and Vitamin D in improving fertility among patients with polycystic ovary syndrome. Clin Exp Reprod Med.

[22] Kamenov Z, Gateva A (2020). Inositols in PCOS. Molecules.

[23] Quach M, Chang YF, Lee I, Tai L, Choi F, Bodemer A (2024). Inositol for treating dermatological disorders: a systematic review. J Integrat Dermatol.

[24] Benelli E, Del Ghianda S, Di Cosmo C, Tonacchera M (2016). A combined therapy with myo-inositol and D-chiro-Inositol improves endocrine parameters and insulin resistance in PCOS young overweight women. Int J Endocrinol.

[25] Nordio M, Proietti E (2012). The combined therapy with myo-inositol and D-chiro-inositol reduces the risk of metabolic disease in PCOS overweight patients compared to myo-inositol supplementation alone. Eur Rev Med Pharmacol Sci.

[26] Lete I, Martínez A, Lasaga I, Centurión E, Vesga A (2024). Update on the combination of myo-inositol/d-chiro-inositol for the treatment of polycystic ovary syndrome. Gynecol Endocrinol.

[27] Antaki R, Desrosiers K, Francoeur D, Magee B, Mills K (2025). Inositol for the Management of Polycystic Ovary Syndrome: SOGC Position Statement. SOGC Position Statement.

[28] Malik N, Dubey N (2021). Role of oral inositol supplementation in women with polycystic ovarian syndrome. Int J Reprod Contracept Obstet Gynecol.

[29] Qamar H, Mustafa R (2020). Role of myo-inositol in treatment of young females affected by polycystic ovarian syndrome: quasi experimental study. J Bahria Univ Med Dent Coll.

[30] Ravn P, Gram F, Andersen MS, Glintborg D (2022). Myoinositol vs. metformin in women with polycystic ovary syndrome: a randomized controlled clinical trial. Metabolites.

[31] Bodepudi R, Seher S, Khan SA (2023). Myoinositol versus metformin in the treatment of polycystic ovarian syndrome: a systematic review. Cureus.

[32] Garg D, Tal R (2016). Inositol treatment and ART outcomes in women with PCOS. Int J Endocrinol.

[33] Laganà AS, Barbaro L, Pizzo A (2015). Evaluation of ovarian function and metabolic factors in women affected by polycystic ovary syndrome after treatment with D-chiro-inositol. Arch Gynecol Obstet.

